# Developing a whole-school mental health and wellbeing intervention through pragmatic formative process evaluation: a case-study of innovative local practice within The School Health Research network

**DOI:** 10.1186/s12889-020-10124-6

**Published:** 2021-01-18

**Authors:** Nina Gobat, Hannah Littlecott, Andy Williams, Kirsten McEwan, Helen Stanton, Michael Robling, Stephen Rollnick, Simon Murphy, Rhiannon Evans

**Affiliations:** 1 Nuffield Department of Primary Care Health Sciences, University of Oxford, Radcliffe Observatory Quarter, Woodstock Road, Oxford, OX2 6GG UK; 2 School of Social Sciences, Cardiff University, Cardiff, UK; 3 Independent Consultant, UK; 4 Health and Social Care, University of Derby, Derby, UK; 5 School of Medicine, Cardiff University, Cardiff, UK

**Keywords:** Process evaluation, Intervention development, Restorative approach, Schools-based intervention, Mental health, Methodology

## Abstract

**Background:**

The evidence-base for whole school approaches aimed at improving student mental health and wellbeing remains limited. This may be due to a focus on developing and evaluating de-novo, research-led interventions, while neglecting the potential of local, contextually-relevant innovation that has demonstrated acceptability and feasibility. This study reports a novel approach to modelling and refining the programme theory of a whole-school restorative approach, alongside plans to scale up through a national educational infrastructure in order to support robust scientific evaluation.

**Methods:**

A pragmatic formative process evaluation was conducted of a routinized whole-school restorative approach aimed at improving student mental health and wellbeing in Wales.

**Results:**

The study reports the six phases of the pragmatic formative process evaluation. These are: 1) identification of innovative local practice; 2) scoping review of evidence-base to identify potential programme theory; outcomes; and contextual characteristics that influence implementation; 3) establishment of a Transdisciplinary Action Research (TDAR) group; 4) co-production and confirmation of an initial programme theory with stakeholders; 5) planning to optimise intervention delivery in local contexts; and 6) planning for feasibility and outcome evaluation. The phases of this model may be iterative and not necessarily sequential.

**Conclusions:**

Formative, pragmatic process evaluations can support researchers, policy-makers and practitioners in developing robust scientific evidence-bases for acceptable and feasible local innovations that do not already have a clear evidence base. The case of a whole-school restorative approach provides a case example of how such an evaluation may be undertaken.

## Background

In recent years there has been a rapid expansion in the number of frameworks available to support the development, modelling and prototyping of complex population health interventions [1, 2]. Despite offering important theoretical, methodological and pragmatic guidance, these frameworks have been largely applied to the development of de novo, research-led interventions rather than to the approaches already in routine practice.

There are distinct benefits of evaluating locally embedded interventions,. First, intervention development frameworks privilege co-production, particularly in regard to developing intervention models that couple stakeholders’ understanding of the problem with scientific evidence. Evaluation of embedded local innovation offers insight into stakeholders’ theorisation of the problem, as they are likely developed in response to these contextually informed understandings. Second, in accordance with realist [3–5] and complex systems perspectives [6, 7], intervention outcomes should be understood as being the result of interactions between the intervention’s causal mechanisms and the context into which they are introduced. In the case of routine practice, much of the dynamic interplay between these mechanisms and context are already emergent. This makes it possible move beyond hypothetical assumptions about how an intervention might operate when introduced to a specific context or how the system will (re) orientate itself following this disruption. Third, acceptability will likely already have transpired, and variations in engagement across different stakeholders may be apparent.

Pragmatic formative process evaluation is an approach that can help the retrospective modelling of locally embedded innovations [8]. It is informed by frameworks used to develop de novo interventions, which tend to include the following research phases: conduct a review to map the nature of the problem and potential intervention responses; establish a stakeholder groups to govern the intervention development process; co-produce intervention materials; test and adapt the intervention in context; and progress to feasibility and/or outcome evaluation [1, 2]. For pragmatic formative evaluations, additional stages will likely need to be considered. These centre on identifying local innovative practice and engaging stakeholders in modelling a programme theory that may have been developed by local practitioners. Transdisciplinary action research (TDAR) approaches have been identified as a way of cultivating and sustaining collaborations to support such additional activities, and may be useful to the pragmatic evaluation approach [9].

Despite their potential value, there is a paucity of empirical examples on how to undertake pragmatic formative process evaluations of complex population health interventions. The present study aims to address this gap by a presenting a worked example. It describes the phases of evaluation undertaken, reflects upon the limitations of the process, recognises the challenges encountered and provides recommendations for the future improvement of the research design. The study draws upon a secondary-school based restorative practice intervention as a case example for testing and developing this approach.

Intervention: The intervention is a system-level approach to restorative practice that has been delivered in a secondary school in Wales since 2008. Restorative practices include relationship-focused actions, which can be implemented at the targeted, universal or whole school level, to impact upon a range of outcomes, including mental health and wellbeing [10, 11]. They often include activities spanning the range of socio-ecological domains (i.e. intrapersonal; interpersonal; organisational; community). A central tenet is to encourage individuals to take responsibility for their actions, with positive engagement in conflict resolution and relationship repair being key to the approach [12]. Key contextual influences that impact on programme theory and implementation practices have not been extensively explored in the existing evidence base. Neither has unintended or potentially harmful causal pathways.

## Methods

A six phased framework was applied to model the intervention and plan potential for further optimisation of delivery and outcome evaluation. These were 1) identification of innovative local practice; 2) scoping review to identify programme theory; contextual characteristics; implementation and outcomes; 3) establishment of a TDAR group; 4) co-production and confirmation of a programme with stakeholders; 5) planning to optimise intervention delivery in local contexts; and 6) planning for feasibility and outcome evaluation. These stages are presented in in detail in the results, with the methodology focusing on the study sample and research methods used.

### Case study

The study comprised case study methodology with one mixed gender secondary school in Wales. The school serves students aged 11–18 years and had more than 1500 registered students in 2016. It has below average student Free School Meal eligibility (FSM) (2016 three-year Welsh average 17.3%), which is routinely used as a proxy measure for socio-economic deprivation. It has an above average proportion of students achieving 5 General Certificate of Secondary Education (GCSEs) at Grade A*-C including English/Welsh and Mathematics (2016 Welsh average 57.9%) [13]. GCSEs are statutory tests taken in Year 11 (age 15–16 years) in England and Wales. The school was identified via the national School Health Research Network (SHRN) infrastructure [14].

### Participant sample and recruitment

Staff and students participated in data generated at Phase 4. The demographic characteristics of participants are presented in Table 1. Students were purposively sampled for maximum variation in gender and age. A total number of 22 students participated. Staff members were similarly sampled to ensure maximum variation, with. Eighteen staff taking part. Staff and students were recruited through the study gatekeeper who was a member of the TDAR group. This individual was a member of staff in the Senior Leadership Team with responsibility for pastoral support, including the school’s implementation of the restorative practice approach. The gatekeeper was asked to purposively recruit participants to ensure diversity.

**Table 1:** Demographic Characteristics of Case Study Participants at Phase 4

	Programme Theory/Logic Model Co-production		Programme Theory/Logic Model Confirmation		Participants who took part in both co-production and confirmation	
	Group 1	Group 2	Group 1	Group 2	***Group 1***	***Group 2***
Students						
**Total students**	**8**	**7**	**8**	**7**	***1***	***7***
Gender						
Male	5	4	5	4	*–*	*4*
Female	3	3	3	3	*1*	*3*
Year group						
Year 7	1	2	2	–	*–*	*–*
Year 8	1	3	3	2	*–*	*2*
Year 9		–	–	3	*–*	*3*
Year 10	1	2	2	2	*1*	*2*
Year 11	2	–	–	–	*–*	*–*
Year 12	2	–	1	–	*–*	*–*
Year 13	1	–	N/A^a^	N/A	*N/A*	*N/A*
Staff						
**Total staff**	**6**	**7**	**8**	**5**	***5***	***4***
Gender						
Male	1	1	–	–	*–*	*–*
Female	5	6	8	5	*5*	*4*
**Role**						
Support staff ^b^	3	1	3	1	*2*	*–*
Teaching staff	1	1	1	2	*1*	*–*
Form tutor	1	1	–	–	*2*	*2*
Leadership role ^c^	1	3	2	2	*–*	*–*
School governor	–	1	–	–	*–*	*–*
Admin staff	–	–	1	–	*–*	*–*

^a^Year 13 no longer at school in July 2016

^b^Support staff members work in the school pastoral support centre

^c^Leadership staff members include heads of year and heads of faculty

### Data collection

Focus groups were selected as the most appropriate method for working with participants, anticipating that interactions would elicit inconsistencies in understandings of the intervention and context. Two focus groups were held with students and two with staff. Researchers used a semi-structured topic guide to steer the discussions (see supplementary files). Focus groups lasted an average of one hour 12 min. Two researchers moderated them. The first set of focus groups were intended to co-produce the programme theory and logic model, while the second set intended to confirm them. An initial, candidate logic model was developed by the TDAR group from the extant research evidence. It was used to start discussion and was built upon throughout the focus groups. The topic guide considered: perceived programme theory; contextual characteristics; experiences of implementation; outcomes; and recommendations for future enhancements. The logic model was refined after the first focus groups and presented at the second set to elicit areas of consensus, areas of non-consensus, and continued uncertainties. Data were generated between April 2016 and July 2016.

### Ethical procedures

The Cardiff University School of Medicine Research Ethics Committee approved the study. All participants were provided with information sheets prior to study commencement, along with the opportunity to ask any questions. Written informed consent was obtained from all participants, with opt-out guardian consent being secured for students.

### Data analysis

Data were audio recorded, transcribed verbatim and reviewed for accuracy. Data collection and analysis were conducted concurrently, with the data from the first set of focus groups being used to inform the questions asked during the second set. Thematic analysis was conducted [15]. Data were initially coded according to the main domains of a logic model (e.g. programme theory; context; implementation; and outcomes). De novo codes were also developed. Coding was undertaken by one researcher and verified by a second. Codes were compared and contrasted to develop themes. The two sets of focus group data were initially considered independently of each other. Themes were then compared across the data to understand changes that emerged through the process of co-producing the programme theory and then confirming it. The final set of themes were confirmed by the wider research team. NVivo10 software was used to support analyses [16].

## Results

**Fig. 1:**
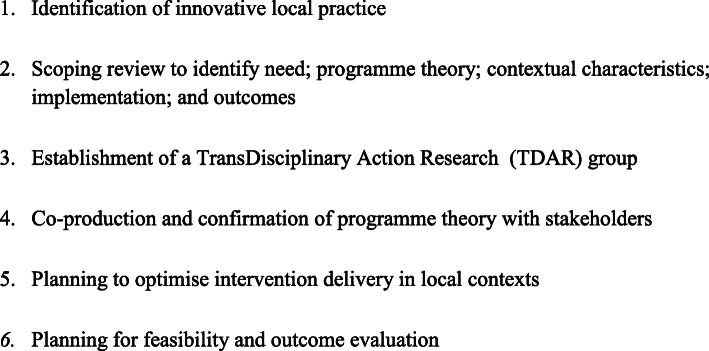
Procedure for conducting the pragmatic formative process evaluation for intervention development and evaluation

The present results describe the six phases of the framework used to identify and model the case of local innovation, in addition to planning to optimise delivery and conduct future feasibility and outcome testing (Fig. 1). These phases are not necessarily sequential and may require repeating a number of times.

### Identification of innovative local practice

The first phase is to identify innovative local practice that warrants progression to modelling and possible outcome evaluation. The researchers identified the case study intervention through the Centre for Development, Evaluation, Complexity and Implementation in Public Health Improvement (DECIPHer) hosted SHRN infrastructure [14]. The network comprised 165 of all secondary schools in Wales (*N*=210) at the time of study, with representation from all 22 local authority areas. SHRN seeks to optimise research collaboration between researchers, policy-makers and practitioners. One of the central mechanisms to encourage collaborative working is through a programme of knowledge exchange activities, including webinars and stakeholder meetings. At regional meetings, researchers present study data whilst practitioners share examples of innovative practice to improve staff and student health and wellbeing. The innovative practice was presented at a stakeholder event, with the school gatekeeper following up the potential for research collaboration with the SHRN Manager. The Manager identified a relevant academic contact with the requisite expertise to assess the fit of the intervention with the centre’s research priorities, formulate preliminary research questions, consider an appropriate research design, and draw together a research team.

Given the characteristics of the local innovation and its history of implementation in the school, a pragmatic formative progress evaluation was decided upon. Criteria for informing this decision was: 1) *Feasibility of programme theory modelling:* The researchers questioned if an “intervention” (regardless of type) was in use and that a programme theory, contextual characteristics, implementation and outcomes could be characterised. The school had been recognised as delivering sector-leading, best practice in restorative practice and had been awarded a Restorative Service Quality Mark (RSQM) in 2010. As a consequence of this external validation the researchers felt that there was clear delivery of a restorative practice intervention. 2) *Feasibility of implementation and scale-up:* The researchers established that the restorative practice had been routinely used and resourced for a substantial period of time (i.e. eight years). The researchers further considered the future traction of the intervention and if it could be scaled-up for evaluation beyond the single case study school, or was so contextually contingent no replication was feasible. There was no indication that the school was atypical so the intervention could not be transported to other secondary schools, and the school had been increasingly invited to share their practices with other schools at a national level due to being recognised as sector leading; *3) Research Co-production:* The researchers consulted with the school to ensure they were prepared to participate in a research study and would potentially be committed to future research.

### Scoping review to identify programme theory; outcomes; contextual characteristics that influence programme theory and implementation

The second phase is to engage in a scoping or systematic review of the existing scientific research to develop a preliminary understanding of the intervention. This can inform the development of a programme theory, which can serve as the basis to model the real-world case example. A review further supports consideration of the effects of such interventions, and potential unintended causal pathways that might be explored in the primary research [12].

#### Causal mechanisms and outcomes

Across the studies there was a lack of specificity around the underpinning programme theory. Rather there were broad principles of how restorative approaches may work, largely through the building, maintaining and restoring of relationships, where individuals take responsibility for their actions and positively engage in relationship repair and conflict resolution [10, 11]. This may be further supported by changes in classroom management practices and school ethos. The INCLUSIVE intervention provides one of the most theoretically informed approaches [12, 17], hypothesising that through restorative practices, students are more likely to engage with schools’ pedagogic practices and embrace rules and ethos. As a result, school connectedness increases and relationships improve. A range of activities at the targeted, universal and whole-school level can be considered as restorative. The approach may be most effective when it is fully adopted at the system level [18, 19].

Evaluations of school based restorative approaches have identified a range of measurable intervention outcomes [12, 18, 20, 21]. At the student level these include improving mental health and wellbeing [12], social and emotional competencies including empathetic attitudes and self-esteem [22], improved academic attainment [21], reduced bullying [22] and fewer school exclusions [20, 21]. There has been limited consideration of staff level outcomes and unintended causal pathways remain largely underdeveloped.

#### Contextual characteristics that influence implementation and programme theory

The researchers mapped key contextual characteristics that might influence the activation of the programme theory and impact planned implementation. The Context and Implementation of Complex Intervention Framework (CICI) [23] was used as a framework for mapping context and implementation. Table 2 shows how the domains of the Framework were populated from the evidence-base. Although existing research findings did not map onto all of the CICI domains, a number of influences emerged across papers. *Epidemiological:* Implementation is strengthened by an increase in the prevalence of bullying within the specified context, leading to more support for such approaches [21, 22]. *Political:* There is increased support for restorative approaches where there is alignment with political/policy priorities, which has often led to direct government funding [12, 18, 20, 24]. *Ethical:* Restorative approaches are congruent with a belief in a fair and just society where citizens are respected. In such cirumstances they are viewed as a more ethical approach to punitative or criminalised responses [20].

**Table 2:** Setting, context and implementation feature of restorative practice interventions from evidence-base [23]

Reference; Study Type	Setting^**a**^	Context^**b**^	Implementation				
			Implementation Theory^**c**^	Implementation Process^**d**^	Implementation Strategy^**e**^	Implementation Agents^**f**^	Implementation Outcomes^**g**^
Bitel (2005) National evaluation report [20]	28 schools (19 restorative approaches & 9 control); mixed urban and rural locations in England and Wales, UK	• *Political:* National commitment to addressing bullying and anti-social behaviour • *Ethical*: Britain values the idea of citizenship. Included in PSHE, part of the educational curriculum	Unclear	Intervention components varied, process of implementation unclear, but involved collaborations with youth offending teams and training	Schools determined the restorative approach they chose to adopt. Senior leadership commitment encouraged	Government funding, youth offending team staff, third sector staff (e.g. Connexions), school staff, students and parents	High levels of staff and student satisfaction with approach. Whole school approach seen as more effective to address antisocial behaviour than partial adoption
Bonell et al. (2015; 2019) Randomised controlled pilot trial; effectiveness trial [12, 17]	8 schools; “satisfactory” or “good” performance as determined by the schools regulatory body (Ofsted) in London and south east England, UK	• *Political:* WHO recognition of bullying and significant impact on adolescent health. British policy context and national initiatives aim to reduce bullying in schools (e.g. 2009 Steer review reported on wide variation in approaches taken by schools to address bullying)	Unclear	Intervention inputs provided and school responsible for implementing these	External facilitator to build commitment among staff, specialist training for staff, training for students	Funding body, external facilitator, school staff and students	Intervention inputs reported as acceptable to staff and students
Kane et al. (2009) [24] McClusky et al. (2008) [18] Pilot evaluation report	18 primary, secondary and special schools; varying rates of exclusion; situated across 3 rural and urban locations with varying degrees of deprivation in Scotland, UK.	• *Political:* Scotland has distinctive social history and educational priorities that draw on humanistic perspectives and sociological understandings of schooling and academic attainment. Most local authorities practice restorative justice to complement Children’s Hearing system. Policy context well aligned with restorative principles, including initiative Better Behaviour, Better Learning • *Ethical:* Recognition that restorative practice is fair and just e.g. approaches advocated	Unclear	Initiation of restorative practice through a government funded pilot scheme. Adaptation to local school needs depending on existing ethos and practice	Training and skill development of school, staff and students	Scottish government; local authorities; school staff and students.	Mixed responses from staff. Some evidence of uptake, but unclear acceptability of implementation processes.
Skinns et al. (2009) [21] Evaluation report	6 schools; mixed gender comprehensives (700–1200 pupils) in South Bristol, UK	• *Epidemiological:* Local: South Bristol location chosen as schools here had the highest rates of exclusion across all schools in Wales and England. Schools described as “problematic”	Unclear	One school integrated approach into school policies and focused on all staff training. Other schools aimed to embed practice in small “pockets”	Training provided for staff	Community interest group, funders, school staff and students	Quality of restorative practice reported to be higher in schools that adopted a whole school approach compared to those that adopted “pockets” of practice. Mixed reception by staff to the model
Wong et al. (2011) [22] Natural experiment	4 secondary schools; equivalent academic attainment records in Hong Kong	• *Epidemiological:* Increase in bullying at school in Hong Kong • *Ethical:* Social preference not to criminalise bullying and aggression in Hong Kong	Unclear	Unclear	All staff trained in a whole school restorative approach	Unclear, but varied. In one school staff and students	One school adopted approach fully, 2 adopted approach partially and 1 did not adopt approach. Unclear how approach was experienced

^a^The specific physical location where the intervention is put into practice; ^b^ Socio-economic, socio-cultural, ethical, legal, political epidemiological, geographical domains; ^c^ Attempts to explain the causal mechanisms of implementation; ^d^ Social processes through which interventions are operationalized in an organization or community; ^e^ Methods and means to ensure the adoption and sustainment of interventions; ^f^ Individuals and organisations engaged with deciding to implement a given intervention, implementing it or receiving it; ^g^ The result or implication of the implementation effort

### Establishment of a TDAR group

The third phase is to establish a TDAR Group, which is intended to support the effective collaboration between diverse stakeholders [25]. The model of TDAR strives for equal, mutually beneficial and reciprocal relationships that value public, practitioner and policy-makers knowledge and experience to the same degree as academic knowledge [26]. Therefore, while dominant terminology uses the term transdisciplinary, it may be more useful to think in terms of creating trans-professional models of practice. t is underpinned by the principles of action research, and its tenets has been increasingly deployed in guidance around intervention development to ensure that approaches are maximally responsive to the contexts where they are to be implemented [1]. Within a pragmatic formative process evaluation, TDAR can help to bring a comprehensive and nuanced understanding of the intervention that is being modelled, in addition to a rich awareness of the context in which it has been originally delivered.

A TDAR group was set up including researchers from different disciplines (i.e. sociology, public health, psychology and epidemiology) and members of the school community who were on the Senior Management Team. The group comprised eight members. It should be noted that students were not represented, meaning that their perspective was only accommodated during the research. Future studies should better represent the target population in the TDAR group. The group met routinely throughout the duration of the study. Its function was to oversee study conduct, ensure that the study design and processes were being shaped by practice perspectives, support the development of a candidate programme theory and to build relationships to support knowledge translation. It further aided the decision-making about future evaluation (Phase 6), where stakeholders could share views on the value of information from an outcome evaluation and the different types of evidence that would support practice moving forward.

### Co-production and confirmation of Programme theory with stakeholders

The fourth phase is the co-production and confirmation of the programme theory, and associated logic model, with key stakeholders to identify the underpinning causal mechanisms, contextual characteristics, implementation practices and outcomes. Participants developed the programme theory through the first round of focus groups. An initial logic model constructed by the TDAR group from the scoping review findings served as a tool to guide this work. The Wisconsin template was used [27]. The output of the logic model is presented in Table 3. A more detailed consideration of context and implementation, as mapped across the CICI framework, is presented in Table 4.

**Table 3:** Logic model for Restorative Practice Intervention

Inputs	Whole school restorative activities	Causal Mechanisms	Intermediate Outcomes	Outcomes
Initiation funding Staff training in restorative approach Policy and systems alignment Benchmarking	**Individual-level** • *Student-staff:* Restorative conversations; Student needs-led approach to learning • *Student –student:* Peer mentoring • *Staff-staff:* Peer mentoring **Group level** • *Classroom*: Circle time; Rotational seating plans • *Staff:* Circle time structure for meetings and policy development **Organisational level** • Distributive leadership • Language of school reflects restorative principles • Student involvement in high stakes school level decisions, e.g. school development planning. **Community level** • Engagement with families • Engagement with local community	Intra-personal skill development – empathy, accountability Enhanced confidence, self-efficacy and sense of achievement in learning among students Enhanced confidence, self-efficacy and reduced stress among staff Trustworthy, supportive, respectful relationships between: • Student-staff • Student-student • Staff-staff Improved relationships between school and families Improved relationships between school and community	School connectedness for students and staff Student engagement in learning and pride in success Positive school culture (e.g. supportive, welcoming, trustworthy, safe and secure) Enhanced school reputation in community and student/staff pride in school	Primary outcome: improved student mental health and wellbeing Improved staff mental health and wellbeing. Increase in student attendance Reductions in student suspension & permanent exclusion. Reduction in staff absence Reduction in students’ referrals to youth justice Reduction in bullying and inappropriate behaviour Improved academic attainment School oversubscription
Contextual characteristics that influence implementation and programme theory				
School level	**Re-enforce and promote cultural shift**	**Undermine or threaten cultural shift**		
	• On-going senior leadership support and investment • Monitoring and evaluation • Self-assessment and development e.g. inset day meetings • Revision of policy documents as active process	• Staff changes and challenge with continuity • Sub-culture of staff resistance and challenge with consistency		
Policy and political level	Contextual drivers that value restorative approach (e.g. the Donaldson review recommending curriculum reform in Wales)	Contextual factors that threaten the approach (e.g. school accountability measures that focus on student results at the exclusion of other metrics)

**Table 4:** Setting, Context and Implementation Features of Restorative Practice Intervention [23]

Setting^**a**^	Context^**b**^	Implementation				
		Implementation Theory^**c**^	Implementation Process^**d**^	Implementation Strategy^**e**^	Implementation Agents^**f**^	Implementation Outcomes^**g**^
Mixed comprehensive, secondary school (1700 students) in Wales. Approx. one quarter of students live in England. Lower than the national average in terms of social deprivation *Interactions* Perception among external stakeholders that restorative practice can work in the school because relatively low social deprivation, with less antisocial behaviour. Also assumption of greater cohesion in family and community groups	*Contextual features* • International: OECD countries compare academic attainment of school students using the Programme for International Student Assessment (PISA) • Regional: Wales score the lowest of UK countries on PISA rankings,. Strong policy focus to enhance academic attainment • Regional: Independent curriculum review in Wales. Recommended changes in approach to attainment and focus on promotion of health and wellbeing • Regional: New legislation in Wales “Well-being of Future Generations Act, 2015” sets legislative frame for public bodies to act in a sustainable way that promotes health and wellbeing. *Interactions* • Embedding of restorative practice as core part of pedagogy aligned with Welsh curriculum review and with new legislative context, but competing pressures regarding academic attainment and school regulatory body targets create opposing tensions and demand • Structures to sustain the intervention requires reflexive practice and adaptability	Diffusion of innovation, where restorative practice initially adoptedby the senior leadership. Recognition that staff and student groups would adopt the intervention at different times and in different ways (e.g. “early” vs. “late” adopters) Theory used to guide and frame experience of implementation over time. Senior leadership use diffusion of innovation terminology to explain process	Implementation process described as “organic”. Started with staff engagement. Moved to re-alignment of school policies and clarification of school values. Transitioned to establishing restorative practice in the form of routines that will sustain the intervention	Funding, training of school staff and students, focus on engagement of innovators and early adopters, use of form tutors to build staff-student class relationship, curriculum review, policy and systems alignment Strategy involves embedding organisational structures that sustain restorative practice (e.g. staff selection, expectation of staff training, the way in which staff meetings are conducted, classroom routines, how the student council is run, expectation of student involvement in high stakes decisions)	Government funding, multi-agency workers, governors, school staff, students and parents	Intervention is fully embedded in the school

^a^The specific physical location where the intervention is put into practice; ^b^ Socio-economic, socio-cultural, ethical, legal, political epidemiological, geographical domains; ^c^ Attempts to explain the causal mechanisms of implementation; ^d^ Social processes through which interventions are operationalized in an organization or community; ^e^ Methods and means to ensure the adoption and sustainment of interventions; ^f^ Individuals and organisations engaged with deciding to implement a given intervention, implementing it or receiving it; ^g^ The result or implication of the implementation effort

#### Causal mechanisms

Both staff and students stated confidence and self-efficacy as being important to the programme theory. Students spoke about feeling equipped to take ownership of their learning, ask for help, and take risks with complex topics, which was largely a consequence of involvement in classroom and school-level decision making. Meanwhile staff suggested that improvements in confidence in the classroom, combined with having the opportunity and skills to express their thoughts and feelings following student conflict, had reduced stress:

*STAFF FG1;3: So … it certainly has made a difference in terms of my wellbeing, giving me more confidence within the classroom … it’s not just looking after student wellbeing, but also staff wellbeing.*

The central mechanism for both of these groups of stakeholders was a change in relationships. Students mentioned peer relationships frequently, while staff emphasised relationships between staff and students: In the later instance, one member of staff suggested that circle time redresses power imbalances, creating more supportive interactions:

STAFF FG2; 14- … the starting with them … with them was to sort of have a circle time in and listen to them. Find out what they need from me and let them know what I need from them. Erm, and just … just not being afraid really to sort of break down any barriers between sort of thoughts and feelings …

Through a shift towards trustworthy and responsive relationships, the school was considered to offer a more positive and supportive culture. These changes led to students experiencing increased school connectedness. This process was further enhanced through a distributed leadership model, involving students in key decision making, such as the design of a new building or appointment of a staff member, with one commenting ‘*we’ve had a huge impact with everything in school*.’

Additionally, students felt that restorative practices had improved the school’s reputation in the community, and relative to other local schools. This had enhanced school connectedness and thus motivation to engage in positive behaviours and improve academic attainment:

STUDENT FG1;5: Because when I first came to the school, … we were known as “down the hill” and now it’s “the comp”. Like things have changed. …

Beyond intended causal pathways, participants considered unintended pathways, which have largely been overlooked in the previous modelling of restorative approaches. This identification illustrates the particular strength of co-production and learning from interventions already in routine practice. For example, participants indicated that the school’s improved reputation following adoption of the intervention had led to over-subscription, which had limited access in the community and placed a resource burden on the school.

#### Outcomes

Participants identified three key sets of outcomes, which are largely congruent with existing restorative approaches. For both students and staff the reported primary outcome was improved student mental health and wellbeing:

STUDENT FG1; 2- I think wellbeing in the school is kind’ve increased massively ..,I’ve got a brother who is 5 years older than me but he came to this school as well and he’s told me stories about how there used to be fights every week and people would set off fire extinguishers... then you look at our school now and honestly I’d be surprised if I heard about a fight because it just doesn’t happen anymore...(laughs) yeah it’s not common any more. I think generally school life has transformed and everything is more positive now. I rarely hear people talk badly about teachers um, everything here seems to be more positive and I think that contributes to all the points these guys have brought up about feeling secure and happy in the environment*.*

Additional outcomes are presented in Table 3.

#### Contextual characteristics

Drawing on the factors identified in the scoping review, the co-production process explored key contextual features that could support the implementation of the intervention and ensure the programme theory worked as intended. These factors were often explained in relation to the reason why restorative practices were initiated.

#### Epidemiological

Data indicated that the school had reached a tipping point, and was ready to change. This was largely was due to perceptions of increasingly poor levels of mental health and wellbeing among students, in addition to high levels of fixed-term and permanent exclusions. Existing practices based on merit and punishment were considered punitive and ineffectual in addressing the problem:

STAFF FG1; 2: … we were just finding we were going round and round and round in circles and not really making progress*.*

#### Political

The policy context in Wales has been increasingly focused on supporting mental health and wellbeing of children and young people, particularly within the educational context. The Well-being of Future Generations Act (2015) in Wales has mandated organisational and culture change to enhance metal health related outcomes. Meanwhile the Donaldson educational review on curriculum reform has outlined six key priorities, such as wellbeing, alongside an acknowledgement of the synergy between wellbeing and educational outcomes (Donaldson, 2016). Although in the case study school, restorative practice had been implemented for 8 years prior to data collection in 2016, and so was in advance of these political and educational changes, these policy priorities support its continued implementation.

#### Socio-economic

Participants acknowledged that the case study school had a lower than national average level of free school meal eligibility and a higher level of academic achievement. Thus, whilst the school cannot necessarily be characterised as atypical, there was acknowledgment that the intervention may be more difficult to implement in a more challenging context with higher levels of disadvantage:

STAFF FG1; 6 – I think there’s more focus on students’ perspectives here um, which students value more. Generally the behaviour here is better than at schools that I’ve taught at previously, though I’d say those schools are working within a different concepts, there are inherently gonna be more issues because of the intake that they have*.*

#### Socio-cultural

Participants suggested that schools tend to have entrenched pedagogic practices that are the antitheses of restorative approaches, namely punitively orientated interactions with students. There is always the risk that staff can orientate to the default approach, which can lead to extensive variation in practice:

STAFF FG1;2 – varied yeah, it is varied across the school: you can see a restorative conversation happening in quite a negative tone in one space, but in another it can be very effective so...and that’s hard for young people as well because young people say “I’ve just had a restorative” (said in an angry voice) and actually it’s like hang on a second, that’s not a restorative

Participants also suggested a potential mismatch between the social and emotional competencies required for the effective delivery of a restorative approach, and a socio-cultural context that does not always privilege vulnerability and emotional openness. To mitigate against such issues, participants identified the importance of senior leadership vision and commitment as part of the implementation plan to ensure realignment of the school ethos with the restorative practice approach and staff commitment to training and delivery. Moreover, the school adopted a rather organic diffusion process, initially securing training to a small team of pastoral staff to ensure their buy in and capacity for modelling the approach before expanding to more diverse professional roles. Eventually working groups were established to ensure continued change to the socio-cultural context, with a Behaviour Research Group reviewing how the restorative practices could be sensitively translated into the setting.

Following the initial round of focus groups to co-produce the programme theory, further work was undertaken by the TDAR to refine their understanding and to create another iteration of the logic model. The second round of focus groups with staff and students was intended to confirm these outputs Importantly they provided clarity on a number of uncertainties that remained following the first round of focus groups and elicited aspects of the intervention and context that had still not been identified. In particular, participants focused on the socio-ecological domains beyond the inter-personal, notably family and community level processes. For example, family-based activities emerged, particularly the delivery of parenting skills, to ensure some congruence between the school ethos and family dynamic:

STAFF FG2;13: We’re working with parents on the approach we would take in school particularly where children have reflected and said ‘well if I did that at home this is what would happe’n … or this is what I see at home. And that ongoing communication and collaboration with parents is really important and it’s quite a long journey for some*.*

Taken together, this phase provided a nuanced and contextually sensitive understanding of the local innovation. At this point it is important to consider the potential for different stakeholder groups to have different perspectives on the programme theory. In the present case example, there were no significant disagreements. However, it may arise and the processes for resolving potential conflict needs further consideration.

### Planning to optimise intervention delivery in local contexts

**Fig. 2:**
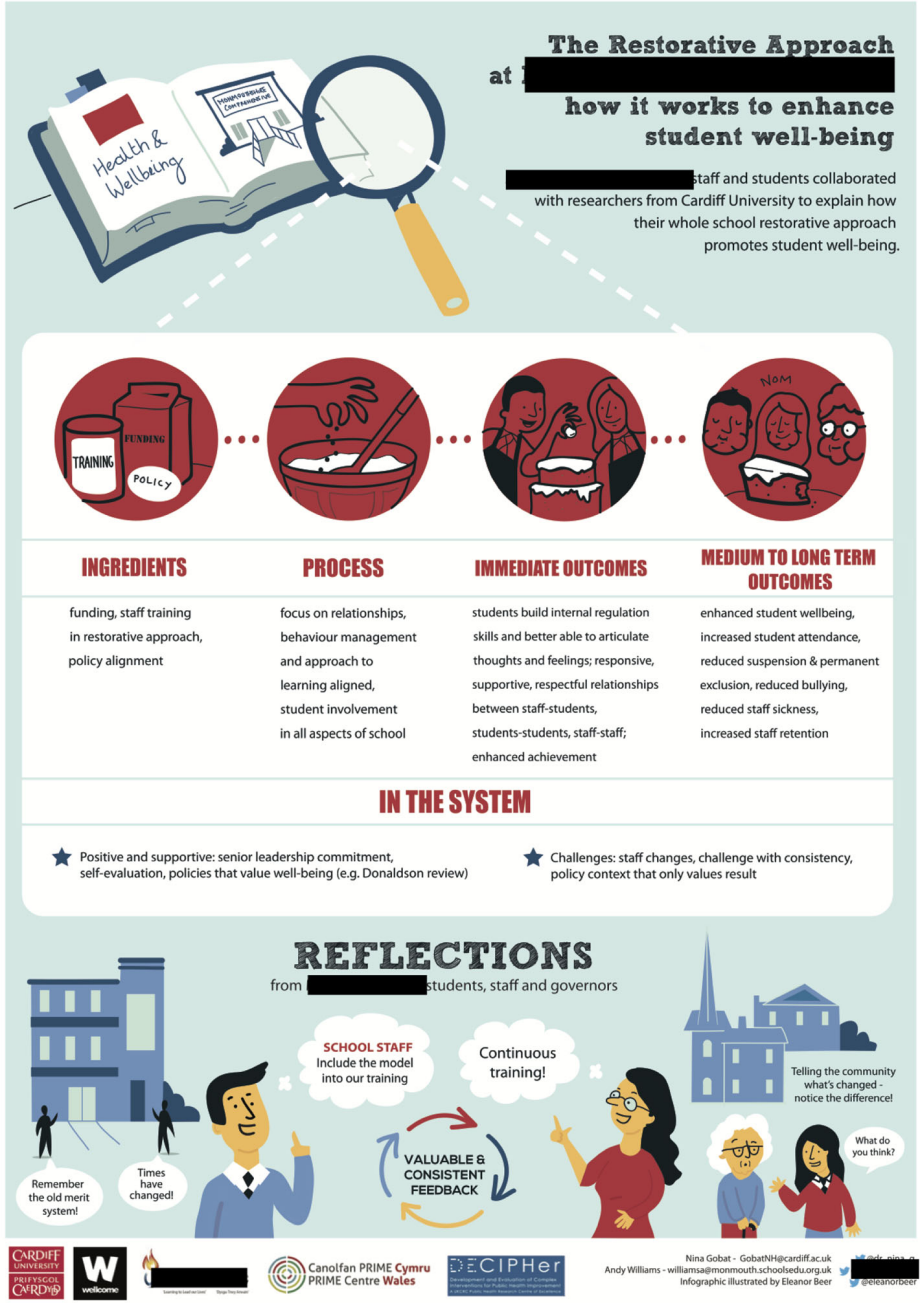
Visual minutes from the whole school restorative approach stakeholder workshop

The fifth phase progresses to planning to optimise the intervention delivery in the local context. A knowledge exchange event was hosted at the school (Fig. 2). The primary purpose of the event was to reflect on the research findings and to identify if there was a mismatch between the hypothetical programme theory that should underpin the approach, and the reality of implementing it within a real-world setting. This was important in exploring if intervention delivery could be optimised to overcome contextual issues that had led to barriers to implementation (e.g. prioritisation of academic achievement), as identified in Phase 4. This is helpful when moving forward to feasibility and outcome evaluation, as it provides some assurance that a future evaluation would not be assessing a sub-optimally delivered approach.

The event also served to address an additional two aims. First, it strengthened partnership between stakeholders. Second, it reasserted the emotional investment of the school [28]. To progress to further evaluation, where the school may be required to support the sharing and delivery of practices within other institutions, it was deemed important for the school feel committed to both the intervention and research. Reflecting with stakeholders provided a positive experience that renewed enthusiasm, with many commenting on how much the school had achieved since the initial introduction of the intervention.

### Planning for outcome evaluation

The sixth phase comprises planning for future outcome evaluation if appropriate. Where outcome evaluation is warranted, the type of evaluation would be most suitably assessed against the phases of evaluation prescribed by the Medical Research Centre (MRC) guidance for developing and evaluating interventions: pilot and feasibility trial, a randomised controlled trial (RCT); natural experiment or other quasi-experimental design; or longer-term implementation evaluation [3, 29]. Further work is required to refine decision-making about the most suitable evaluation approach, and an a priori progression criteria similar to that used in feasibility trials may be helpful to guide next steps after the pragmatic formative process evaluation. Potential criteria to be considered are: 1) The evaluability of the intervention [30]; 2) The Value of Information (VOI), which weighs the cost of obtaining evidence against the need for certainty amongst stakeholders [31]; and 3) the applicability of the existing evidence base to the local context. For example, Aarons et al. have developed a framework for ‘borrowing evidence’, which assesses the similarities of different interventions and contexts to see if the outcomes of evaluations conducted elsewhere have relevance to the new context in question [32].

In the present case study, planning is primarily being conducted through the TDAR group. The SHRN infrastructure offers a particular opportunity to continue with pragmatically orientated innovation evaluation, through the conduct of a pragmatic feasibility and outcome trial. As of 2020 the network includes 100% of the 210 state-funded schools in Wales, providing a complete sample frame for randomisation. A sample of students at each participating school complete bi-annual surveys of their health and wellbeing, and provided data is collected at appropriate times, these surveys could be exploited as the data source for outcome measurement. As popular innovations, such as that selected for the case study, are gaining traction within systems, it is imperative that we have responsive study designs. Use of routine data, such as that collected through the SHRN survey data offers such responsivity, although the evidence generated is arguably less scientifically robust than that provided by RCTs.

## Discussion

In recent years there has been a proliferation of guidance on the development of complex population health interventions [1, 2]. Such frameworks have primarily focused on the modelling of de-novo interventions. To date there has been more limited consideration of the retrospective development of local innovations that are already routinised. Such approaches offer a fruitful opportunity in population health improvement. In recent years there has been growing interest in the idea that intervention effectiveness is contextually contingent. In response, a range of theoretical and methodological tools are being developed to help anticipate how context may impact upon an intervention’s functioning [23, 33, 34]. Yet in the event of routinised practice, many of these contextual contingencies are already emergent or even established. This may make it easier to implement them in comparison to novel approaches, as potential barriers and facilitators may be understood.

The case study intervention, a school-based restorative practice approach addressing student mental health and wellbeing, demonstrates the utility of pragmatic formative process evaluations. To date there have been a range of restorative interventions, including that reported in the recent INCLUSIVE trial [12, 17]. While many of these studies have started to map key system influences that may moderate the intervention’s programme theory, the case study is particularly insightful as it presents established contextual characteristics eight years into intervention implementation. These include key socio-cultural factors, such as the entrenched educational ethos and pedagogic approaches [35]. Such findings also illustrate the importance of attending to intervention maintenance, and the ongoing resource required to ensure continued contextual fit. Use of context mapping frameworks, such as the CICI framework, across studies reporting on different phases of diffusion will enable researchers to understand the evolution of contextual factors and how interventions may respond to and accommodate them [23].

The six phases of intervention modelling are particularly focused on the elicitation of contextual characteristics. To this end, meaningful co-production must serve as a central feature. As with other developmental frameworks, establishment of TDAR group is recommended to ensure that a diverse range of stakeholders invested in the intervention are adequately represented [2, 25]. The presence of this group can help ensure that phases of evaluation privilege co-production, that policy and practice stakeholders are able to make a meaningful contribution and that the modelled intervention captures a multiplicity of experiences and perspectives.

Pragmatic formative process evaluation also responds to the ever-present issue of the mismatch between the needs of policy-makers and practitioners and the reality of conducting scientifically robust evaluations. One of the key tensions is the timeliness of generating research evidence, and a perceived lack of responsiveness in the research community. Efforts to resolve these arguably incompatible needs have increasingly focused on quasi-experimental designs, with natural experiments being used to evaluate policy innovation [36]. While such designs may not provide the same level of scientific robustness as randomised controlled trials, they do allow for the generation of pragmatic and relevant evidence. The present framework for pragmatic formative process evaluation supports this direction of travel by engaging the wealth of local innovation that has already gained traction within real world settings, rather than prioritising new approaches largely developed by researchers.

### Limitations

There are a number of limitations that should be acknowledged. First, as with existing developmental models focused on co-production, it is uncertain how much stakeholders should contribute to gain a nuanced understanding of the intervention [2]. The proposed phased approach risks privileging researchers’ perspectives by commencing with the review and synthesis of existing literature. Equally the phases of stakeholder engagement may be inadequate in practice, and they may need to be continually repeated until the logic model is fully refined and there is consensus. Second, the representativeness of the case study school should be considered, as it had a lower than average level of free school meal eligibility, a higher than average level of academic attainment and a large student population. The field of implementation has been increasingly concerned with the generalizability of evidence when interventions are scaled-up or scaled-out [32], and there are considerations about whether the intervention could be embedded within schools of different socio-economic profiles. For example, study participants felt it would be challenging to deliver the intervention in more socio-economically deprived settings, while extant research suggests that the quality of staff-students relationships is actually more of a priority in schools of a lower socio-economic status [37]. Third, while maximum variation in sampling within the case study was pursued, the sample is limited by those who were prepared to participate. Focus groups were largely conducted with students engaged in classroom level activities, and did not include many individuals who had received one-to-one support. Equally, data were not available on additional student level characteristics that may have influenced perceptions of the intervention (e.g. school connectedness) and these were not addressed during recruitment. Fourth, the composition of the student focus groups, which were heterogenous in gender and school years, may have inhibited the sharing of contrasting views and encouraged students to conform to predominant norms.

## Conclusion

The present study provides an empirically worked example of a pragmatic formative process evaluation to support researchers, policymakers and practitioners in the modelling, delivery and outcome evaluation of interventions already in routine practice. This phased framework serves as a complement to the emerging range of guidance for the development of de-novo population health interventions [1, 2], by addressing the specific developmental phases required for working with locally embedded innovation. It also responds to increased policy and practice needs, where evaluation needs to be responsive to the rapid emergence of new innovation. Further methodological and empirical work is needed to apply and refine the framework with different health outcomes, populations and settings.

## Data Availability

The datasets generated and/or analysed during the current study are not publicly available due to protection of participant privacy and confidentiality but are available from the corresponding author on reasonable request.

## Abbreviations

CICI: Context and Implementation of Complex Intervention framework
DECIPHer: Centre for Development, Evaluation, Complexity and Implementation in Public Health Improvement
FSM: Free School Meal
GCSE: General Certificate of Secondary Education
INCLUSIVE: intervention - Initiating change locally in bullying and aggression through the school environment intervention
MRC: Medical Research Centre
RCT: randomised controlled Trial
RSQM: Restorative Service Quality Mark
SHRN: School Health Research Network
TDAR: Transdisciplinary Action Research
VOI: Value of Information
